# High-dose remifentanil exacerbates myocardial ischemia-reperfusion injury through activation of calcium-sensing receptor-mediated pyroptosis

**DOI:** 10.7150/ijms.83207

**Published:** 2023-09-18

**Authors:** Yejing Zhu, Jinyu Chi, Shunv Cai, Siqi Liu, Junbo Yuan, Hongliang Xu, Huidan Zhou

**Affiliations:** 1 Department of Anesthesiology, Zhejiang Cancer Hospital. Hangzhou, Zhejiang Province, 310022, China.; 2 Laboratory of Anesthesia and Perioperative Medicine, Hangzhou Institute of Medicine (HIM), Chinese Academy of Sciences, Hangzhou, Zhejiang 310018, China.; 3 Department of Cardiology, First Affiliated Hospital of Harbin Medical University, No. 199 Dazhi Street, Harbin, 150001, China.

**Keywords:** High-dose remifentanil, CaSR, Myocardial ischemia-reperfusion, Pyroptosis, Reactive oxygen species.

## Abstract

**Background:** The aim of this study was to investigate whether calcium-sensing receptor (CaSR) was involved in HRF-mediated exacerbation of MI/R injury through NLRP3 inflammasome activation and pyroptosis.

**Methods:** *In vivo*, a rat MI/R model was established by ligating the left coronary artery, and short-term HRF exposure was induced during reoxygenation. Then, TUNEL, H&E, Masson staining, immunohistochemical (IHC) and serum levels of lactate dehydrogenase (LDH) and creatine kinase isoenzyme (CK), as well as the expression levels of CaSR and pyroptosis-related proteins in heart tissues, were measured. H9c2 cells were cultured to create a hypoxia/reoxygenation (H/R) model and exposed to different concentrations of RF. After pretreatment with the CaSR activator gadolinium chloride (GdCl_3_) and inhibitor NPS2143 in the H/R model and treatment with HRF, we compared cellular viability, TUNEL, cytosolic [Ca^2+^]_i_, the levels of LDH and CK, pyroptosis-related proteins and CaSR in H9c2 cells. We further researched the mechanisms of CaSR-mediated pyroptosis in the H/R+HRF model by CaSR-shRNA, Ac-YVAD-CMK, MCC950 and NAC.

**Results:** We found that HRF significantly increased CaSR expression, rate of cell death, levels of CK and LDH, and exacerbated pyroptosis in MI/R model. *In vitro*, HRF increased CaSR expression, decreased viability, enhanced cytosolic [Ca^2+^]_i_ and exacerbated pyroptosis in H/R cells. Pretreated with GdCl_3_ worsen these changes, and NPS2143, MCC950, Ac-YVAD-CMK, NAC and sh-CaSR can reversed these effects.

**Conclusion:** Exposure to HRF for a short time exacerbates MI/R-induced injury by targeting CaSR to increase cytosolic [Ca^2+^]_i_ and ROS levels, which mediate the NLRP3 inflammasome and pyroptosis.

## 1. Introduction

Remifentanil (RF), a μ-receptor opioid agonist, has been widely used as an analgesic for general anesthesia 1. With its short-acting effect and rapid dissipation, RF enables the administration of high doses of opioids within a short period of time during prolonged surgeries. Cardioprotection of RF has been demonstrated in myocardial ischemia-reperfusion (MI/R) by several studies. Some studies suggest that small amounts of RF protect the myocardium against hypoxia/reoxygenation (H/R) injury by enhancing autophagic flux or reducing lipid peroxidation 2, 3. Also, small amounts of RF can effectively reduce myocardial cell injury caused by myocardial ischemia reperfusion in rats, improve cardiac function, decrease cleaved caspase-3 and increase the ratio of Bcl-2/Bax in myocardial cells 4. Many researches focused on the protective effects of RF in MI/R, while ignoring its side effects in high doses. Thus, whether such acute exposure to high dose short-acting opioids induces a detrimental change in cellular oxidative state is not known and the molecular mechanisms need to exposed.

As we known, MI/R is a complex pathological process, including pathological injury of the myocardium mediated by oxidative stress and excessive accumulation of reactive oxygen species (ROS) 5. Some studies have showed short term exposure to HRF significantly increases myocardial superoxide production with concomitant enhancement of oxidative stress in MI/R, and corrupts the heart's sensitivity to be preconditioned by opioids 6. HRF can also enhance oxidative stress and cause secondary damage to the cells, as seen from the elevation of 8-OHdG in myocardium 6. However, the mechanism by which HRF aggravated MI/R injury remains unclear. Previous study reported that oxidative stress accompanied by the generation of ROS could be observed, and ROS played a key role in stimulating the inflammatory reaction by activating the NOD-like receptor pyrin domain-containing protein 3 (NLRP3) pyroptosis pathway 7. Increasing evidence suggests that inflammation, including proinflammatory cytokines such as interleukin (IL), plays a critical role in MI/R injury 8, 9. Among the various inflammasomes, the NLRP3 inflammasome plays an important role in inflammatory responses and pyroptosis and mediates the maturation of the proinflammatory cytokine IL-1β by activating caspase-1 10, 11, 12. Interestingly, the NLRP3 inflammasome can be activated by diverse stimuli called danger-associated molecular patterns (DAMPs) although calcium-sensing receptor (CaSR) 13.

CaSR is a member of the superfamily of G protein-coupled receptors (GPCRs) and is functionally expressed in the myocardium, parathyroid gland, kidneys, bone, and vessels 14. Our previous studies showed that CaSR participates in apoptosis and inflammation in myocardial cells 15, 16. Some studies have also revealed that CaSR regulates the NLRP3 inflammasome through calcium (Ca^2+^) and cAMP 13. However, to the best of our knowledge, no study has reported whether CaSR is recruited in HRF-exacerbated myocardial cell pyroptosis. It is also uncertain whether HRF aggravated MI/R injury through directly inducing pyroptosis.

In this study, we established a rat MI/R model with HRF to examine whether HRF aggravates MI/R injury via CaSR-mediated NLRP3-pyroptosis. In addition, H9c2 cells were exposed to hypoxia/reoxygenation and treated with HRF during the reoxygenation to verify the underlying mechanisms.

## 2. Materials and methods

### 2.1 Materials

Antibodies against CaSR (19125-1-AP), GSDMD (20770-1-AP), Caspase-1/P20 (22915-1-AP), and IL-18 (10663-1-AP) were purchased from Proteintech (Proteintech Group, Inc, USA), antibodies against NLRP3 (A5652) and IL-1β (A1112) were purchased from ABclonal (Wuhan, China). Antibodies against ASC (sc-22514R) were purchased from Santa Cruz Biotechnology (Santa Cruz, CA, USA). Antibodies against GAPDH and all secondary antibodies were obtained from Absin (Shanghai, China). Horseradish peroxidase (HRP)-conjugated anti-mouse IgG and anti-rabbit IgG were purchased from Cell Signaling (Beverly, MA, USA). NPS-2143 (HY-10171) and Gadolinium chloride (GdCl_3_) (HY-103314) were purchased from MCE (New Jersey, USA). Ac-YVAD-CMK(C4810) and MCC950(B7946) were purchased from Apexbio (Houston, Texas, USA). NAC was purchased from Beyotime Biotechnology (Shanghai, China).

### 2.2 Animals and Myocardial Ischemia‒Reperfusion Model

Animal experiments were approved by the Animal Ethics Committee of Cancer Hospital of University of Chinese Academy of Science (Zhejiang Cancer Hospital). Male Sprague‒Dawley rats (250-300g) were housed in separate cages and given food and water except before the study and exposed to 12 h light and dark cycles. Then, the rats were randomly divided into three groups: sham, MI/R and HRF+MI/R. An open chest rat model was used, and the methods have been described in detail previously 6. In brief, mice were anesthetized by the intraperitoneal injection of 1% pentobarbital sodium (80 mg/kg) and then underwent endotracheal intubation to receive mechanical ventilation from an animal ventilator. A left thoracotomy was performed to expose the heart, and a 6-0 Prolene loop, along with a snare occluder, was placed at the origin of the left coronary artery. Thirty minutes of regional ischemia was induced by pulling the snare and securing the threads on the left anterior descending branch; then, we untied the ligature and began reperfusion for 2 h. With this approach, MI/R injury was eventually induced. In the HRF + MI/R group, remifentanil infusion was administered at a dose of 20 μg/kg/min during reoxygenation, which was described in detail previously.

### 2.3 Histological Analysis and Immunohistochemical Staining

The paraffin-embedded rat myocardial tissue was sectioned to 5 µm. H&E staining and Masson staining in heart sections were carried out according to the manufacturer's instructions as described in a previous study. Paraffin-embedded heart sections (5 μm) were prepared. Then, sections were incubated with anti-CaSR antibody (1:100 dilution) at 4°C overnight. Subsequently, the sections were incubated with horseradish peroxidase (HRP)-conjugated secondary antibody for 30 min at room temperature. Fresh diaminobenzidine (DAB) solution was added to the sections, which were counterstained with hematoxylin. Images were captured under a microscope.

### 2.4 Terminal deoxynucleotidyl transferase dUTP nick end-labeling (TUNEL) immunofluorescence

*In vivo*, after paraffin-embedding fixation, each heart was sliced at the slagged area. According to its manual, the one-step TUNEL immunofluorescence detection kit from Beyotime (Shanghai, China) can measure DNA fragments *in vivo*. *In vitro*, a TUNEL immunofluorescence detection kit (Beyotime, Shanghai, China) was used to detect the death of cardiomyocytes in each group after the corresponding treatment was administered. Fluorescent images were acquired with a confocal laser scanning microscope (Olympus FV1000, Japan).

### 2.5 Cell culture and treatments

Embryonic rat heart-derived H9c2 cells (iCell Bioscience Inc, Shanghai, China) were cultured in DMEM (Gibco-BRL, Gaithersburg, MD) supplemented with 10% fetal bovine serum (FBS), streptomycin (100 µU/ml) and penicillin (100 µU/ml) and grown in a humidified 5% CO_2_ incubator at 37°C. Stock cultures were passaged at 2- to 3-day intervals. Cells were incubated in 6-well plates (2 × 10^5^ cells/well) or 96-well plates (1 × 10^4^ cells/well) for different experiments. Then, H9c2 cells were subjected to H/R, as described in a previous study. Briefly, cells were cultured in serum- and glucose-free DMEM in a hypoxic chamber (Adelbio, Clermont-ferrand, France) saturated with a gaseous mixture of 1% O_2_, 5% CO_2_, and 95% N_2_ at 37°C for 5 h. After hypoxia, the cells were reoxygenated for 4 h by incubation under normoxic conditions in serum-free DMEM containing glucose. Control normoxic cultures were also prepared. For experiments, H/R cells were stimulated with different concentrations of RF for different reoxygenation time. The CaSR agonist GdCl_3_ (30 μM) was added and incubated for 15 min, and the CaSR inhibitor NPS2143 (10 μM) was added 60 min before HRF administration.

### 2.6 Cell Counting Kit-8 viability assay

A Cell Counting Kit-8 (CCK-8; Beyotime, China) was used to measure cell viability according to the manufacturer's instructions. H9c2 cells were seeded in 96-well plates at 1 × 10^4^ cells/well. At the end of the treatment period, 20 μl of CCK-8 solution was added to the cells and incubated in the dark for 1 h at 37°C. The absorbance of each well was measured at 450 nm relative to the reference absorbance at 630 nm using a microplate reader (Bio-Rad, USA).

### 2.7 Measurement of culture supernatant levels of CK, LDH, caspase-1, IL-1β and IL-18 activity

Culture supernatants were collected, and CK and LDH activities were determined to evaluate cell injury by using commercially available CK and LDH assay kits (Jiancheng, Nanjing, China) according to the manufacturer's instructions. Caspase-1 activity was assayed by using a caspase-1 activity assay kit (Beyotime, Shanghai, China) according to the manufacturer's instructions. The concentrations of IL-1β and IL-18 were assessed by enzyme-linked immunosorbent assay (ELISA) (Elabscience, China) according to the manufacturer's instructions.

### 2.7 Electron microscopy

Electron microscopy was used to determine the occurrence of pyroptosis. As described previously, cells were seeded in 6-well plates, treated as indicated, fixed in 2.5% glutaraldehyde in Hank's modified salt solution, postfixed in 1% OsO_4_ in 0.1 M cacodylate buffer, scraped off and dehydrated in a series of ethanol solutions. Dehydration was completed in propylene oxide, and the specimens were embedded in Araldite. Ultrathin sections were prepared with an ultramicrotome, mounted on copper grids, and contrasted with lead citrate. Specimens were analyzed and documented by electron microscopy.

### 2.8 Detection of ROS

Intracellular ROS levels were detected with a ROS detection kit (Beyotime, China). The main components of the kit were 2′,7′-dichlorodihydrofluorescein diacetate (DCFH-DA), which has no fluorescence. DCFH-DA can freely flow through the cell membrane and generate 2′,7′-dichlorodihydrofluorescein (DCFH) in the cell under the effect of the cell ester hydrolysis enzyme, which cannot freely pass through the cell membrane. DCFH can be oxidized in the presence of ROS to generate the fluorescent substance 2′,7′-dichlorofluorescein (DCF). The level of green fluorescence is directly proportional to the intracellular ROS level. The fluorescence intensity of intracellular ROS was recorded using a fluorescence microscope (Olympus FV1000, Japan).

### 2.9 Fluo-4/AM Measurements of Intracellular [Ca^2+^]_i_

Cells were cultured in confocal culture dishes and then loaded with 2 mM Fluo-4/AM for 30 min at 37°C in the dark, washed twice with Ca^2+^-free PBS to remove the extracellular Fluo-4/AM and incubated further in DMEM. Changes in [Ca^2+^]_i_ were measured by the fluorescence intensity induced by Fluo-4 in cells recorded using confocal scanning microscopy (Olympus FV1000, Japan).

### 2.10 Immunofluorescence analysis

Cells were exposed to HRF (based on electron microscopy and immunoblotting results) and fixed in 4% paraformaldehyde for 20 min at room temperature. All remaining procedures were completed at room temperature, except when otherwise noted. After being washed for 10 min in PBS, the cells were permeabilized with a 0.5% Triton X-100/PBS solution for 30 min and then incubated for 20 min in 5% goat serum to saturate nonspecific binding sites. The cells were incubated with monoclonal antibodies against CaSR (1:100) and NLRP3 (1:100) overnight at 4°C. The next day, the cells were washed twice in PBS and incubated with tetramethylrhodamine isothiocyanate-conjugated secondary antibodies diluted in PBS (1:50) for 1 h at 37°C. Fluorescent images were acquired with a confocal laser scanning microscope (Olympus FV1000, Japan).

### 2.11 Western blot analysis

Briefly, cells were washed twice with ice-cold PBS and incubated in lysis buffer containing the protease inhibitor phenylmethylsulfonyl fluoride for 30 min. The cells were centrifuged at 12,000 rpm for 15 min at 4°C to remove the nuclei and undisrupted cells. The protein concentration of the supernatant was determined using a BCA protein assay (Beyotime, Shanghai, China), with bovine serum albumin (BSA) as a standard. Total protein (30-50 μg) was subjected to SDS‒PAGE (8% (w/v) acrylamide gel) and blotted onto PVDF membranes in 39 mM glycine, 48 mM Tris (pH 8.3), 20% (v/v) methanol, and 10% SDS at 200 mA for 90 min in a water-cooled transfer apparatus. The membranes were blocked in Tris-buffered saline plus Tween (TBS-T) (137 mM NaCl, 20 mM Tris [pH 7.6], and 0.1% [v/v] or Tween 20) containing 5% (w/v) 5% BSA at 37°C for 1 h. The membranes were incubated overnight at 4°C with antibodies against CaSR (1:1000), NLRP3 (1:1000), ASC (1:1000), Gasdermin-D (1:1000), IL-18 (1:1000), IL-1β (1:500), and caspase 1 (1:1000). The membranes were incubated with HRP-conjugated anti-IgG antibodies (1:5000) in TBS-T for 1 h at 37°C. Antibody-antigen complexes were detected using ECL, and ImageJ (v.1.34 17, 18, NIH) was used to quantify the bands.

### 2.12 Lentiviral infection of H9c2 cells

H9c2 cells were seeded in 12-well plates according to specific experimental requirements. After 24 h, the cells were infected with lentiviruses (Tsingke Biotechnology, Beijing, China) containing CaSR-shRNA or Con-shRNA at a multiplicity of infection (MOI) of 20. Cells were incubated with the lentiviruses for up to 72 h to allow maximal transfection. Then, the cells were harvested and analyzed by western blotting to confirm the knockdown efficiency of CaSR. Cells were then subjected to H/R and treated with HRF during reoxygenation.

### 2.13 Statistical analysis

All data are expressed as the mean ± standard deviation (SD). Statistical analyses were performed with SPSS version 26.0 software (SPSS, Inc., Chicago, IL, USA), and plots were drawn by GraphPad Prism 8.3 software (GraphPad Software, Inc., San Diego, CA). Statistical differences were assessed by one-way analysis of variance (ANOVA) for multiple groups. Bonferroni's post-hoc test was used to test all pairwise comparisons between groups. Values measured repeatedly at different time points within a group were tested by one-way analysis with repeated measurements. Differences were considered statistically significant at *p < 0.05, **p < 0.01 and ***p < 0.001.

## 3. Results

### 3.1 HRF exacerbates myocardial injury by motivating CaSR and pyroptosis in MI/R myocardium *in vivo*

**Figure 1:**
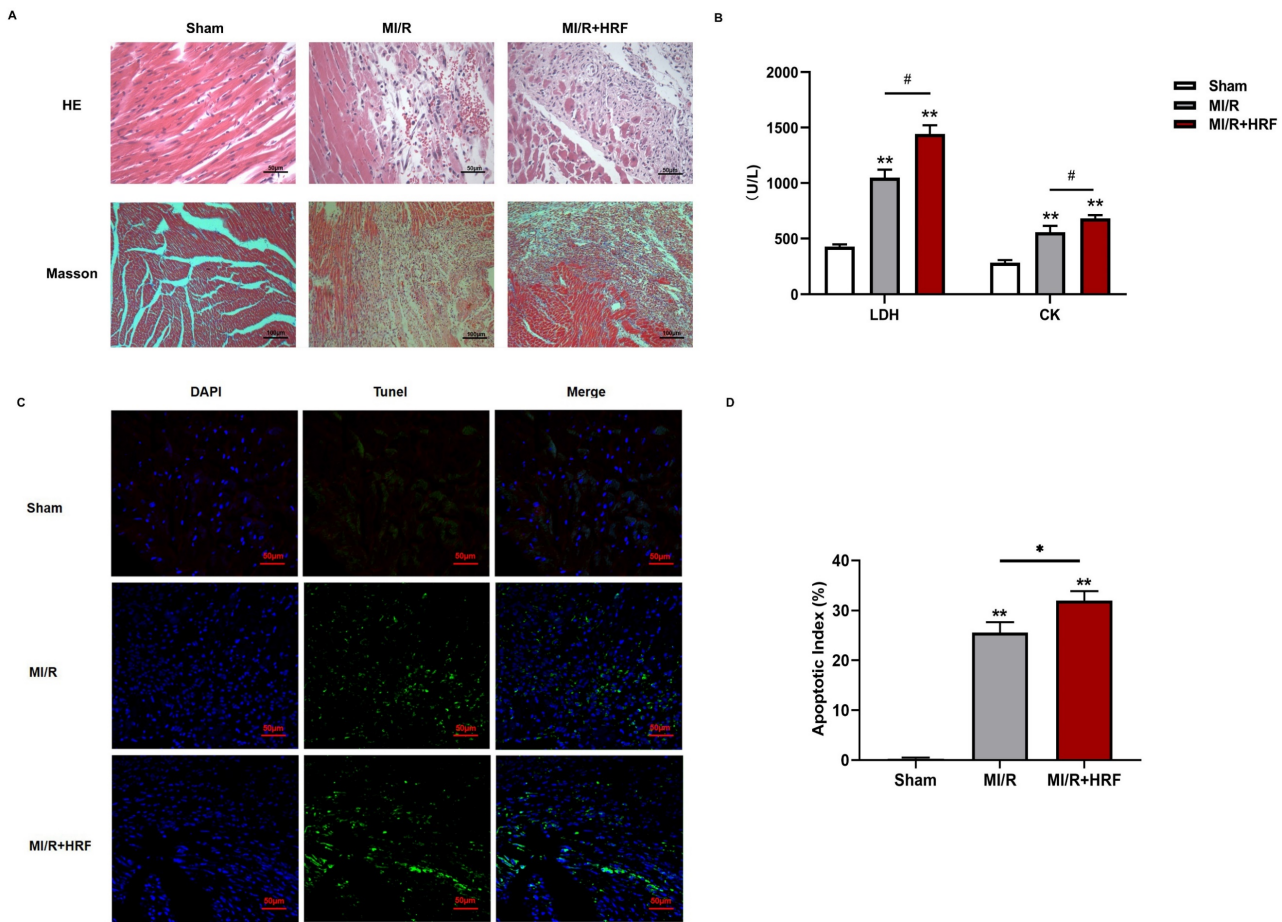
HRF exacerbates myocardial injury in MI/R myocardium *in vivo*. **(A)** H&E staining and Masson staining of myocardial tissues in three groups (n=5 per group); **(B)** Comparison of serum LDH and CK in three groups (n=5 per each group); **(C)** Results of Tunel immunofluorescence (n=5 per each group); **(D)** statistical comparison of apoptotic index *p < 0.05 and ** p < 0.01 versus sham. #p<0.05 versus MI/R

**Figure 2:**
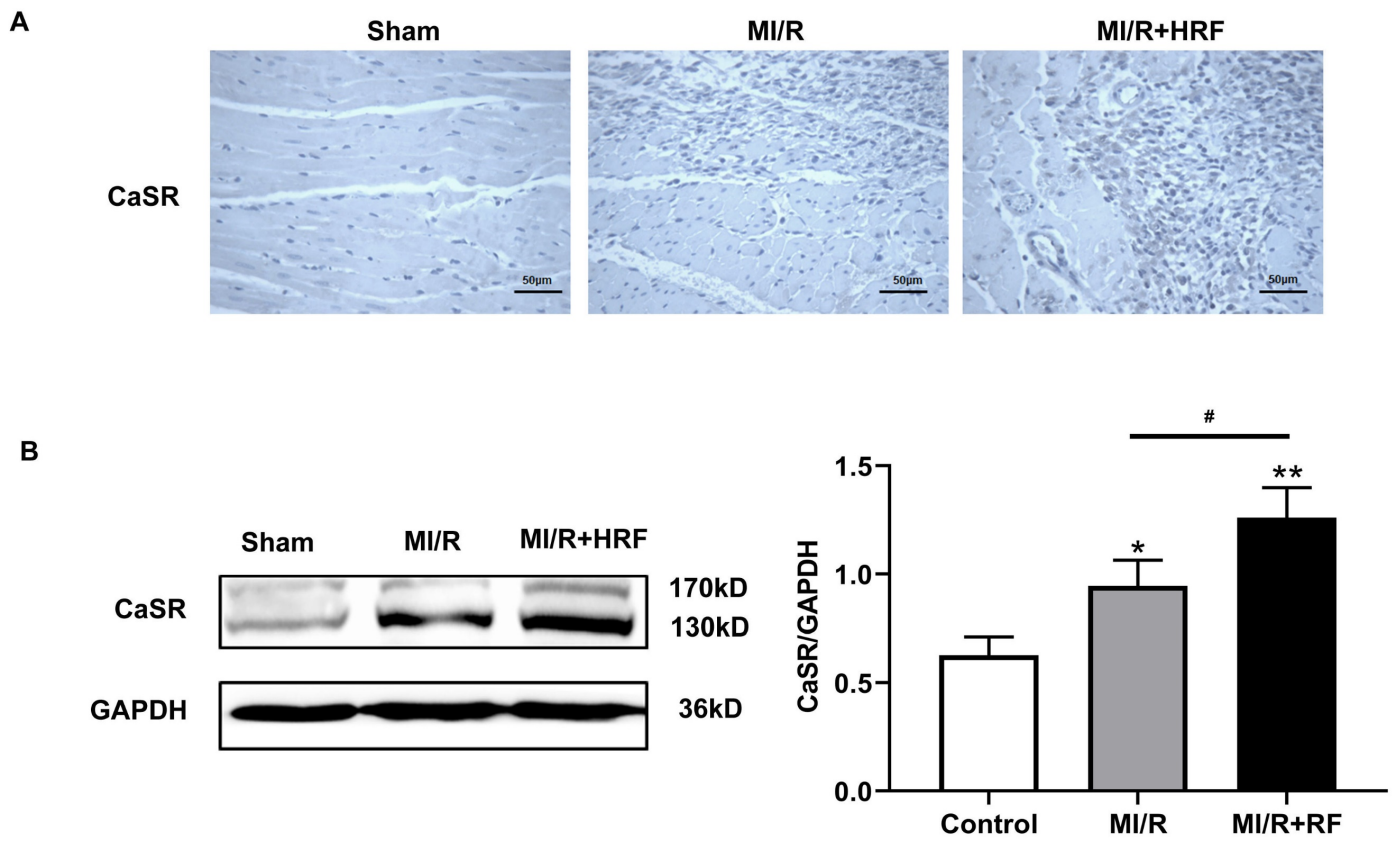
HRF exacerbates myocardial injury and increased CaSR expression. **(A)** Immunohistochemical staining was used to detect CaSR in heart tissues in three groups.** (B)** Western blotting assay was used to detect the protein level of CaSR. **p < 0.01 versus Sham group. #p<0.05 versus MI/R.

**Figure 3:**
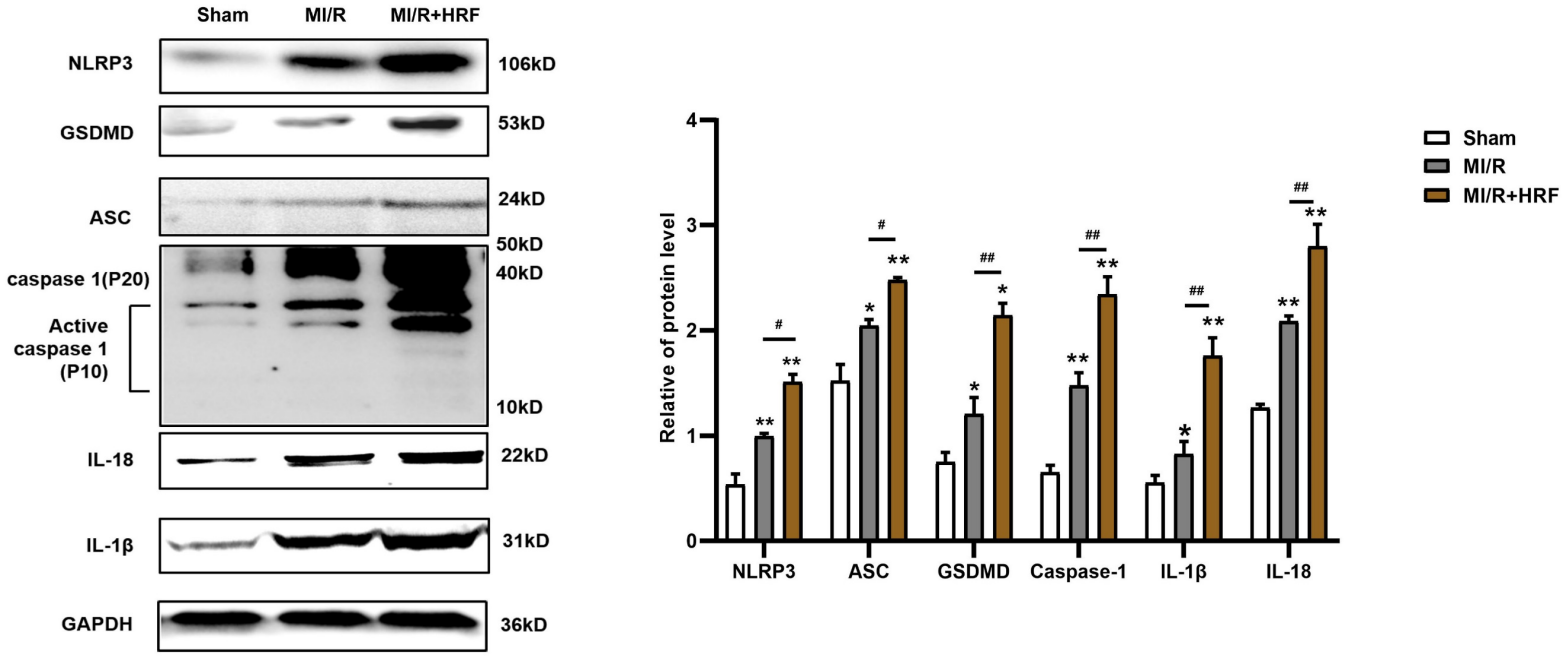
Increased NLRP3 inflammasome and pyroptosis-related proteins in HRF-exacerbated MI/R injury. Western blotting assay was used to detect the protein level of NLRP3, ASC, caspase-1, GSDMD, IL-1β, and IL-18 in heart tissues in three groups. GAPDH was used as a load control. **p < 0.01 versus Sham group. #p<0.05 versus MI/R.

The levels of serum CK and LDH were significantly higher in the rats of the MI/R + HRF group relative to the rats in the other two groups (Fig. 1B), which indicated that the HRF-aggravated MI/R model was successfully created. Moreover, H&E and Masson staining were conducted in the myocardium to observe the effect of HRF on cardiac structure after MI/R injury. For the MI/R and HRF+MI/R groups, obvious cardiac injury was demonstrated (damaged myocardial fiber structure, disorganization of the muscle fibers, inflammatory cellular infiltrations and interstitial edema). As shown by Masson staining, the degree of myocardial fibrosis was obvious in the MI/R and MI/R + HRF groups (Fig. 1A). Cardiomyocyte death in the MI/R + HRF groups was significantly higher than that in the other two groups (Fig. 1C). In comparison to sham mice, increased CaSR expression was found in MI/R tissues, which was even more obvious in the HRF+MI/R group (Fig. 2A). Meanwhile, compared with the MI/R group, the expression levels of CaSR, NLRP3, ASC, caspase-1, GSDMD, IL-1β, and IL-18 were significantly higher in the HRF+MI/R group (Fig. 2B and 3).

### 3.2 HRF exacerbates H/R injury in H9c2 cardiomyocytes

**Figure 4:**
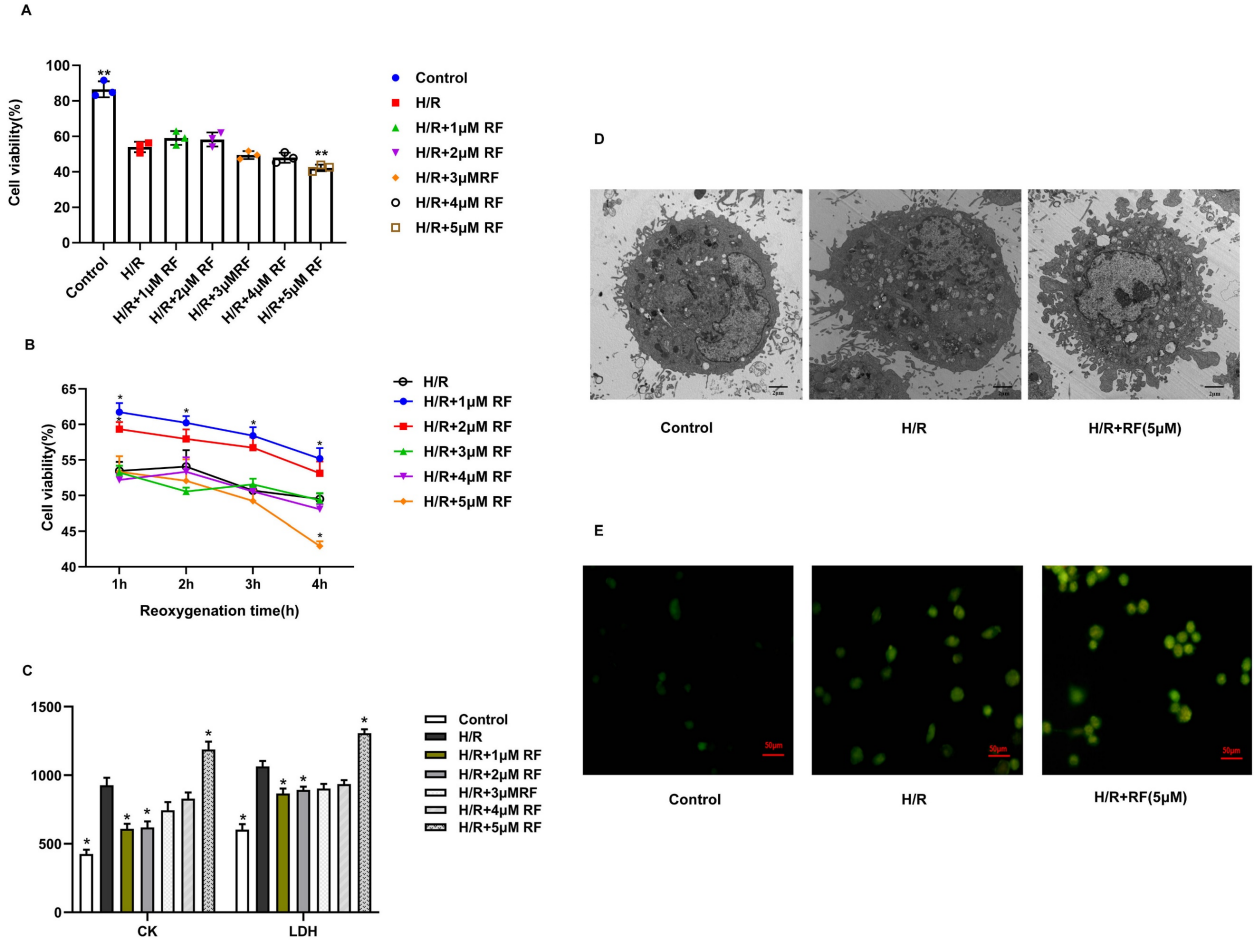
Effects of different doses of RF in different reoxygenation time on H/R cells. **(A-B)** Cell viability was measured by the CCK-8 assay. **(C)** LDH and CK release was measured respectively. The results are the average of three independent experiments performed in triplicate. *p<0.05 versus the H/R group; #p<0.05 versus the control. **(D)** Transmission electron microscopy showing pyroptosis in the H/R and H/R+RF groups. H9c2 cells were subjected to H/R and stimulated with 5 μM RF for 4 h. The cells were fixed and analyzed by electron microscopy (original magnification ×8000).** (E)** Changes in the intensity of fluorescence of [Ca^2+^]_i_ were recorded with a laser scanning confocal microscope under different treatment conditions.

To determine the effect of RF on the activity of H/R cells, we used CCK-8 assays to confirm the effects of different concentrations of RF on the viability of H/R-injured cells during different reoxygenation time (Fig. 4A-B). After H/R stimulation, H9c2 cells showed a massive decrease in viability compared to those in the control group. The viability of H9c2 cells recovered in the presence of 1 μM RF and declined in response to 2-4 μM RF. Increasing the dose of RF (5 μM) obviously decreased H9c2 cell viability after H/R. Then, we measured the levels of CK and LDH in the supernatant after H/R-injured cells were stimulated with different doses of RF. The results showed that the 5 μM RF group exhibited higher levels of CK and LDH than the H/R or control groups (Fig. 4C). Moreover, as shown in Fig. 4D, transmission electron microscopy showed that H9c2 cells gradually became swollen and mitochondria were malformed after H/R. After stimulation with 5 μM HRF, fragmentation of the cell membrane and swollen mitochondrial structures were obviously observed in H/R-injured cells. Then, we measured the levels of cytosolic [Ca^2+^]_i_ and observed an increase in cytosolic [Ca^2+^]_i_ in H/R cells. This effect was enhanced when H/R cells were treated with HRF (Fig. 4E). These results suggest that HRF exacerbates H/R injury by increasing [Ca^2+^]_i_.

### 3.3 HRF-induced exacerbation of H/R injury in H9c2 cardiomyocytes was associated with increased CaSR and NLRP3

**Figure 5:**
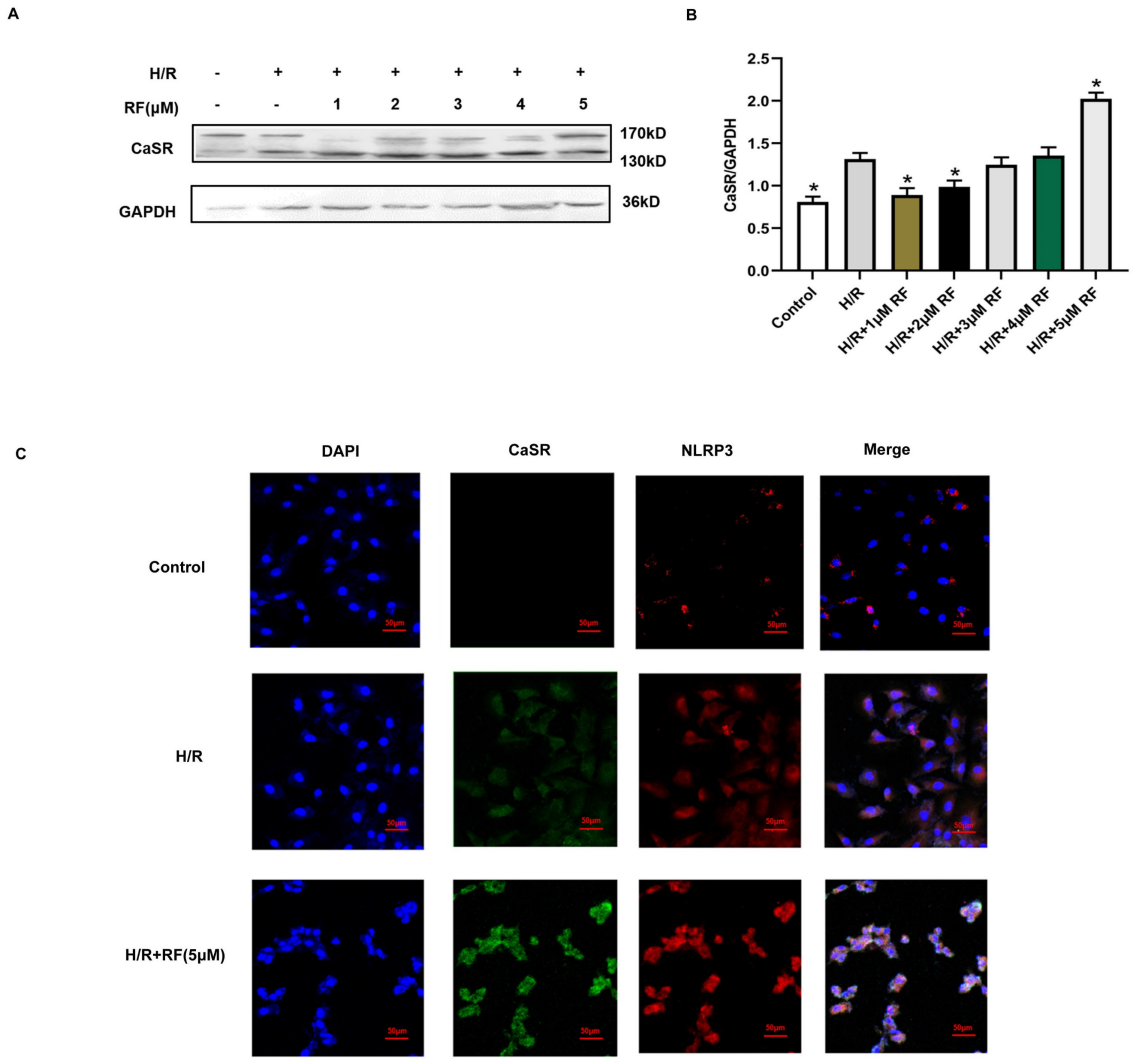
RF treatment affected the expression of CaSR and activation of NLRP3-associated pyroptosis under H/R conditions. **(A)** Western blot analysis showing CaSR expression in response to various RF concentrations. **(B)** The data show the densitometric analysis of CaSR relative to GAPDH levels. The results are the average of three independent experiments performed in triplicate. *p<0.05 versus the H/R group; #p<0.05 versus the control. **(C)** H9c2 cells were subjected to H/R and exposed to 5 μM RF for 4 h, and then the localization and expression of CaSR and NLRP3 were analyzed by immunofluorescence.

CaSR is associated with intracellular calcium release. We then examined changes in CaSR protein expression in response to different doses of RF in H9c2 cells stimulated with H/R. The results showed that compared with cells in the control group and H/R group, H/R-injured cells exposed to HRF exhibited increased levels of CaSR (Fig. 5A-B). According to the above findings, the chosen dose of RF and exposure time resulted in increased CaSR and NLRP3 expression, as shown by an immunofluorescence assay. Dramatic and robust cytoplasmic accumulation of CaSR and NLRP3 was observed throughout the cell in the HRF+H/R group (Fig. 5C).

### 3.4 HRF exacerbates H/R-induced cell injury through CaSR-mediated activation of NLRP3 and upregulation of the Caspase-1 pathway

**Figure 6:**
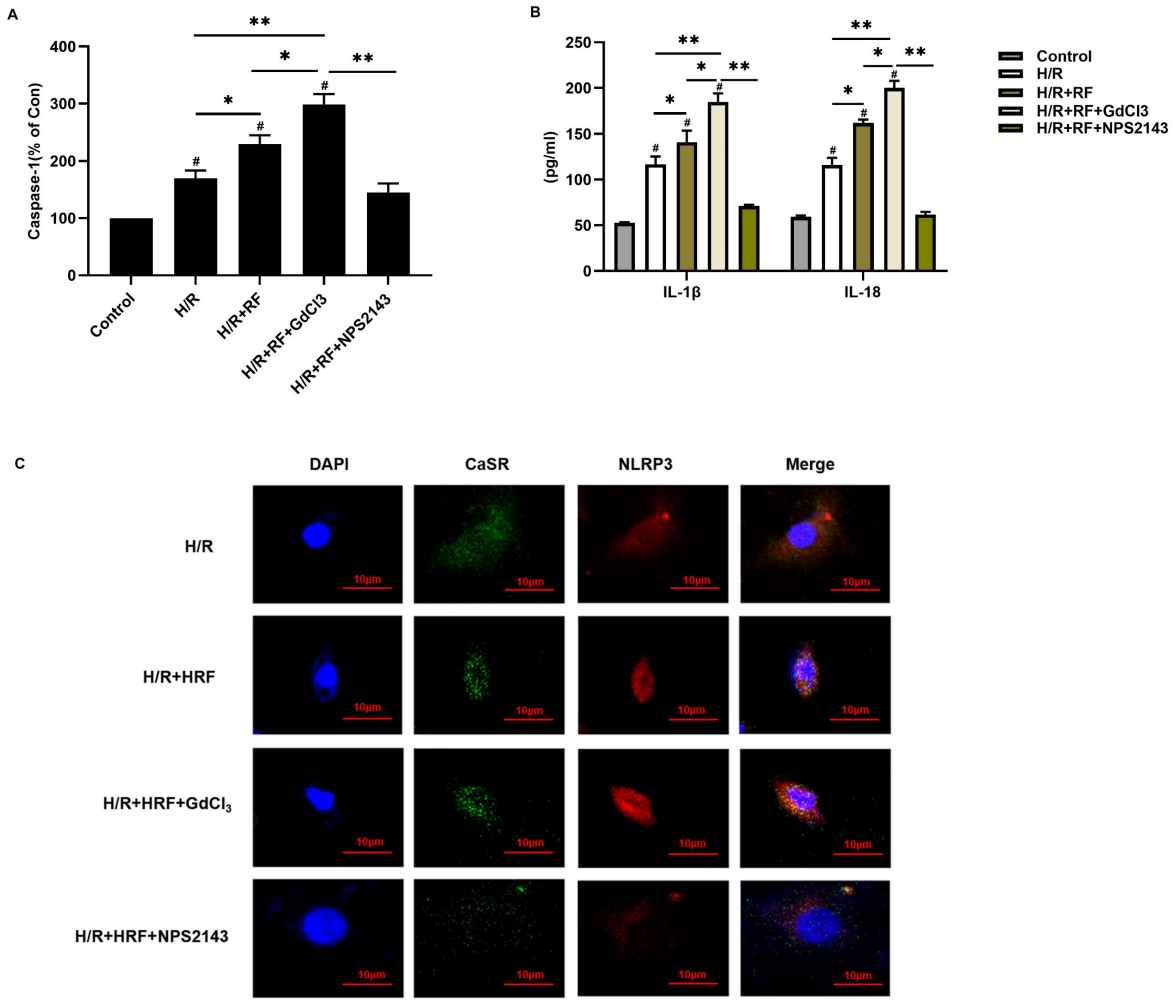
CaSR mediated activation of the NLRP3 inflammasome and enhanced caspase-1 activity and IL-1β and IL-18 release in H9c2 cells under H/R and H/R+RF conditions.** (A)** Caspase-1 activity was measured by a caspase-1 activity assay kit.** (B)** The levels of IL-1β and IL-18 in the supernatants were analyzed by ELISA. The results are the average of three independent experiments. # p<0.05 versus the control; *p<0.05, **p<0.01.** (C)** Immunofluorescence of CaSR and NLRP3 with GdCl3 and NPS2143 under H/R and H/R+RF conditions.

To investigate the role of CaSR in HRF-induced pyroptosis, H9c2 cells stimulated with H/R were pretreated with the CaSR activator GdCl_3_ or with the CaSR inhibitor NPS2143 and then exposed to HRF. We measured caspase-1 activity and IL-1β and IL-18 levels by ELISA in the supernatants. Our results revealed that caspase-1 activity was significantly increased in the H/R and H/R+HRF groups compared with the control group and was even higher in the H/R+HRF+GdCl_3_ group than in the H/R+HRF group. However, pretreatment with the CaSR inhibitor NPS2143 impeded this process in H/R-injured cells (Fig. 6A). Consistent with the change in caspase-1 activity, the levels of IL-1β and IL-18 in the H/R group and H/R+HRF group were increased compared with those in the control group and were further increased in the H/R+HRF+GdCl_3_ group compared with the H/R+RF group. Meanwhile, as shown by immunofluorescence, increased CaSR and NLRP3 expression in the cytoplasm was revealed significantly in the H/R+HRF+GdCl_3_ group, while NPS2143 markedly blocked the effect of HRF on H/R-injured cells (Fig. 6B-C).

**Figure 7:**
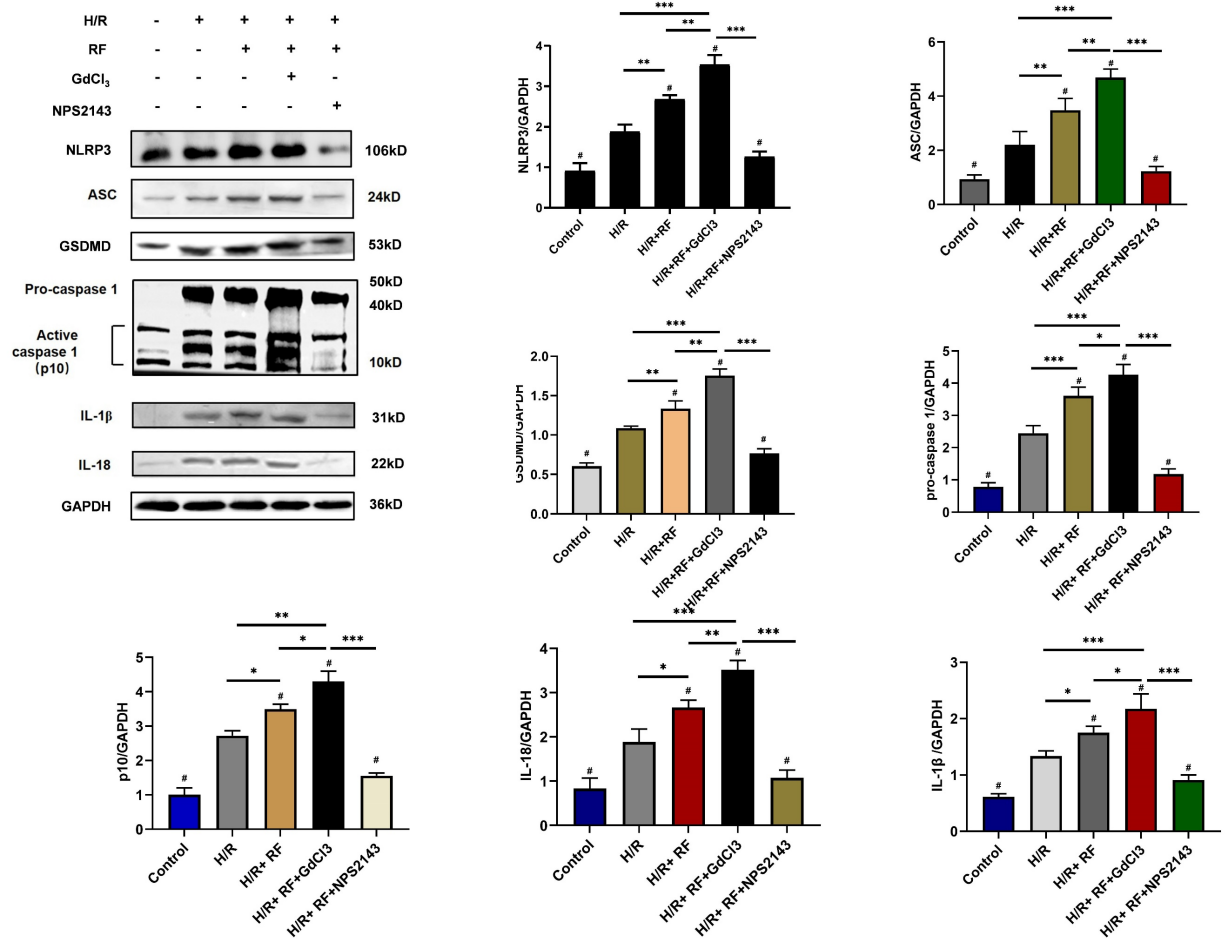
HRF enhances pyroptosis via activation of CaSR and upregulation of the caspase-1 pathway following H/R injury. H/R-injured cells were pretreated with GdCl3 or NPS2143 and then exposed to HRF for 4 h. Western blot analysis showing the expression of NLPR3, ASC, GSDMD, procaspase-1, active caspase1 (p10), IL-18 and IL-1β. The data show the densitometric analysis of the target proteins relative to GAPDH levels. Data are presented as the mean ± SD. # p < 0.05 versus H/R. *p<0.05, **p<0.01, ***p<0.001.

To identify potential pathways responsible for these changes, we measured the expression of ASC, GSDMD, procaspase-1, active caspase-1 (p10), IL-18 and IL-1β in these groups. As shown in Fig 7, compared with the control group and the H/R group, the HRF+H/R group exhibited higher protein expression of ASC, GSDMD, procaspase-1 (p20), active caspase-1 (p10), IL-18 and IL-1β. GdCl_3_ significantly increased the protein expression of ASC, GSDMD, pro caspase-1 (p20), active caspase-1 (p10), IL-18 and IL-1β in HRF-treated H/R-injured cells. In contrast, NPS2143 reversed the increased expression of these proteins.

### 3.5 Knockdown of CaSR inhibits pyroptosis induced by HRF in H/R-injured cells

**Figure 8:**
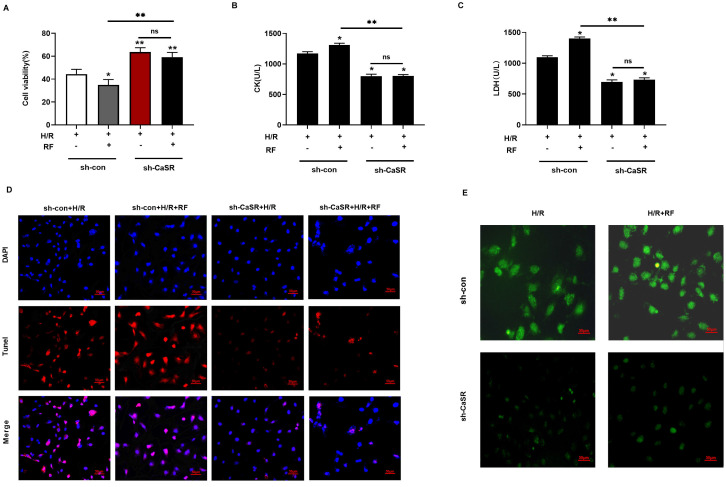
Knockdown of CaSR alleviated HRF-exacerbated myocardial injury under H/R condition. **(A-C)** shRNA to silence CaSR expression and investigated the effects on cell viability, CK and LDH. *p<0.05 versus shCon. ** p<0.01. **(D)** Results of Tunel immunofluorescence in four groups. **(E)** Detection of intracellular ROS levels.

**Figure 9:**
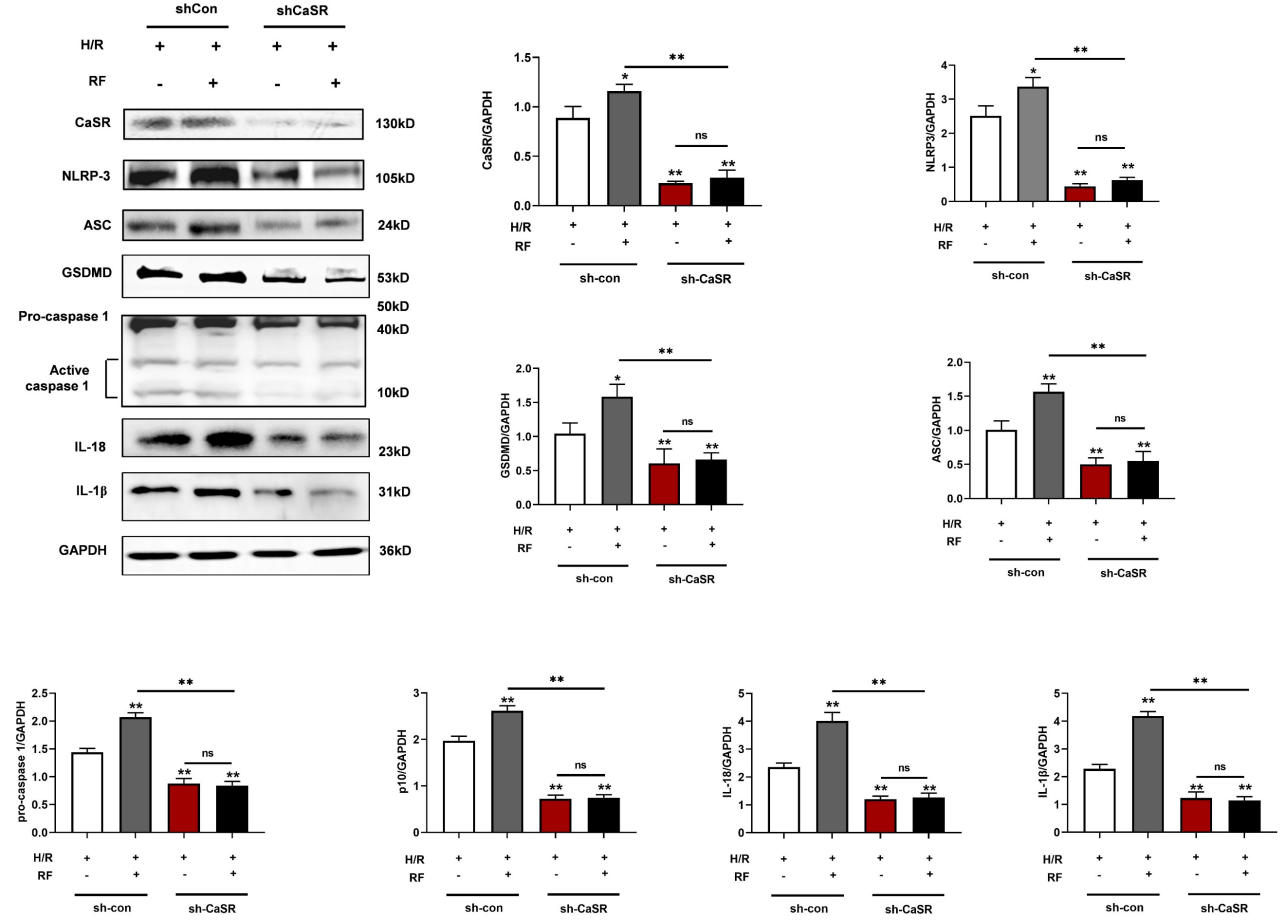
Knockdown of CaSR inhibited pyroptosis-related proteins in H/R cells with or without HRF. Western blot analysis showing the expression of CaSR, NLRP3, ASC, GSDMD, procaspase-1, active caspase1 (p10), IL-18 and IL-1β. The data show the densitometric analysis of the target proteins relative to GAPDH levels. The results are the average of three independent experiments performed in triplicate. * p<0.05 versus the H/R group. ** p<0.01.

To further verify the role of CaSR in the caspase-1 pyroptosis pathway induced by HRF, we used specific shRNA to silence CaSR expression and investigated the effects on cell viability, CK and LDH. As shown in Fig 8A-C, there were significant increases in cell viability and the levels of CK and LDH compared with those of shCon cells (Fig 5A-5C). As shown in Fig 8D, treatment with shCaSR evidently suppressed pyroptotic cell death compared with that of shCon cells by TUNEL. Compared with the H/R group, HRF stimulated more ROS production in shCon cells, and yet ROS production was decreased in shCaSR cells even with HRF stimulation (Fig. 8E). Moreover, CaSR, NLRP3, ASC, GSDMD, pro-caspase 1 (p20), active caspase-1 (p10), IL-1β and IL-18 were decreased in shCaSR cells compared with shCon in H/R-injured cells with HRF-induced exacerbation of pyroptosis, as previously demonstrated by Western blotting (Fig. 9).

**Figure 10:**
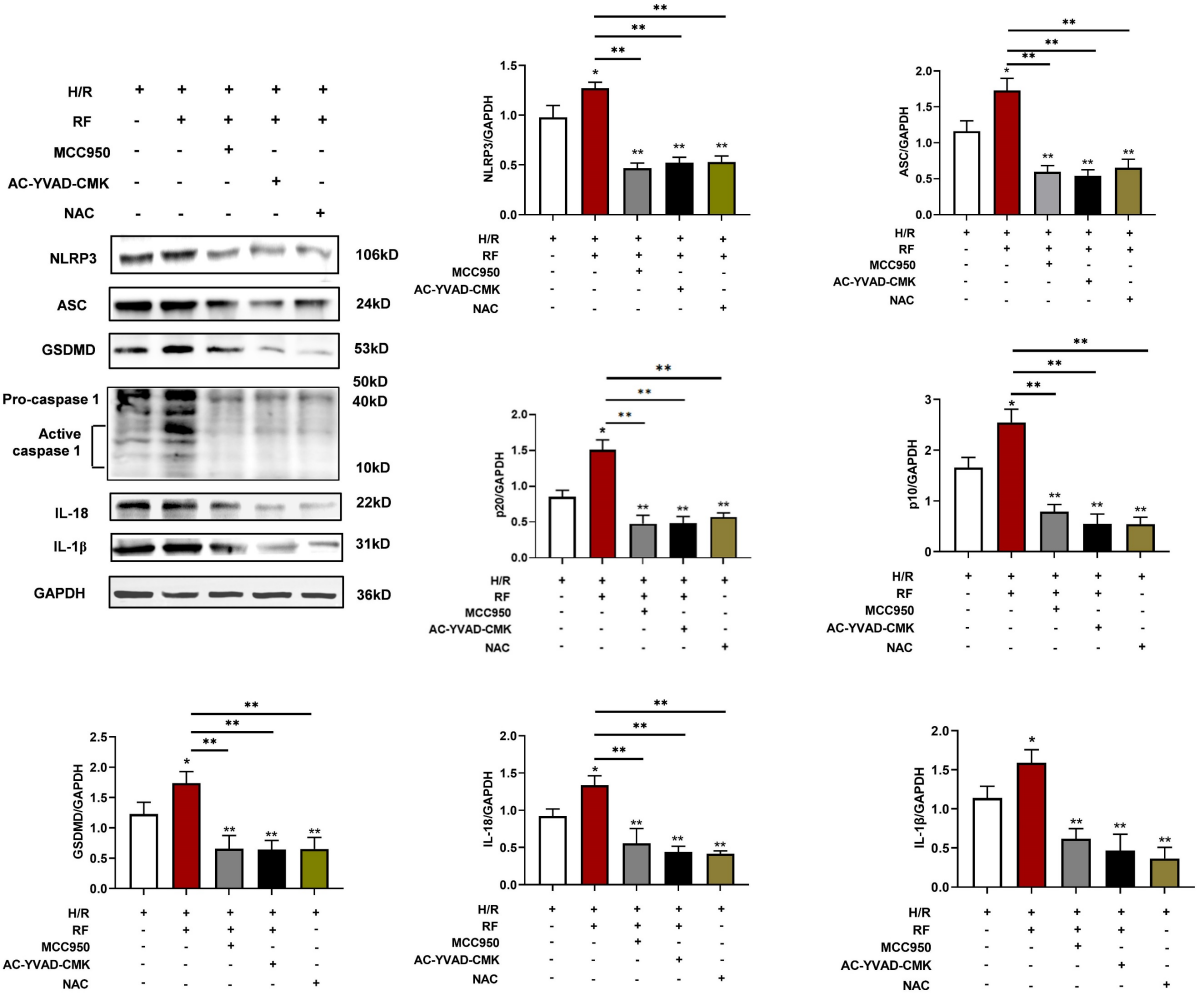
Expression levels of pyroptosis-related protein. Cells were pretreated with MCC950, Ac-YVAD-CMK, and NAC at the onset of oxygenation. Western blot analysis showing the expression of NLRP3, ASC, GSDMD, procaspase-1, active caspase1 (p10), IL-18 and IL-1β. The data show the densitometric analysis of the target proteins relative to GAPDH levels. * p<0.05 versus the H/R group. ** p<0.01.

To determine the effects of pyroptosis pathways on HRF-exacerbated H/R injury-related signaling, cells were pretreated with MCC950 (an NLRP3 inhibitor) at 10 μM, Ac-YVAD-CMK (a caspase-1 inhibitor) at 50 μM for 30 min, and NAC (an ROS scavenger) at 10 mmol/L for 1 h at the onset of oxygenation. As shown in Fig. 10, MCC950 and Ac-YVAD-CMK markedly blocked the effect of HRF on H/R injury in H9c2 cells by downregulating pyroptosis-related protein levels. Treatment with NAC also showed the above effect. Our data indicated that activation of CaSR-mediated pyroptosis pathways is involved in HRF-exacerbated H/R injury.

## 4. Discussion

In this study, we found that HRF exacerbated MI/R injury by increasing CaSR- and pyroptosis-related protein expression. CaSR activation causes intracellular calcium release, further pyroptosis activation and increased ROS production in myocardial cells. However, suppressing CaSR and NLRP3-related pyroptosis signaling could block this process. These findings showed that activation of CaSR-mediated pyroptosis is involved in HRF-exacerbated MI/R injury* in vitro* and *in vivo*.

RF is a μ-opioid receptor agonist that is widely used in clinical anesthesia and has been reported to have dual effects on the myocardium. The different concentrations of remifentanil may trigger cardioprotection or enhance myocardial injury. Studies have shown that RF postconditioning (RPC), which used a low concentration of RF (1μM), significantly reduces myocardial infarction in mice following MI/R *in vitro* 17, 18. On the other hand, some studies have suggested that HRF leads to severe cardiovascular depression 19. To date, most investigations of RF have focused on RF postconditioning (RPC), which can provide myocardial protection against ischemic insult 20, 21, only a few studies have shifted attention to the negative effects of HRF on the heart. However, HRF-induced negative myocardial effects cannot be ignored, and it is critical to understand the mechanisms. Due to the short half-life of RF, it was important to determine whether it is the concentration of the drug or the duration of exposure that may be responsible for any observed differences. In our study, we successfully created HRF-aggravated MI/R mouse model. We found that the rats that underwent MI/R had elevated levels of CK and LDH, which are the main markers of myocardial damage. When HRF (20 μg/kg/min) stimulated MI/R rats, CK and LDH showed further increases, more serious cardiac injury and gradual myocardial fibrosis were displayed by H&E and Masson. This finding reconfirmed that HRF aggravated MI/R injury in previous studies 6. Some studies have reported that the TUNEL-positive cell rate and caspase-1 are widely used to detect pyroptosis 7. In HRF+MI/R rats, the apoptotic index (AI) and the expression of caspase-1 increased more significantly than those in MI/R rats. These results suggest that HRF-aggravated MI/R injury is associated with pyroptosis. *In vivo*, the results showed that a low concentration of RF (1-2μM) increased cell survival following H/R injury, whereas a high concentration of RF (5μM) exacerbated H/R-induced injury in H9c2 cells by decreasing viability and increasing CK and LDH levels. As the exposure time increases, the protective effect of the low concentration of RF diminishes, while the injury caused by HRF gradually escalates. These results were consistent with previous findings that different doses of RF have opposing effects on the myocardium.

Many studies have shown that CaSR is expressed throughout the cardiovascular system, such as in the myocardium 22. Previously, we have shown that upregulation of CaSR expression can induce cardiomyocyte calcium overload, which is related to apoptosis and autophagy in cardiomyocytes and fibroblasts 15, 23. Furthermore, CaSR plays an important role in a variety of physiological and pathological conditions, such as MI/R 24. Thus, we hypothesized that CaSR was involved in HRF-mediated exacerbation of MI/R-induced injury. Evidence from this study supports our hypothesis. *In vivo*, the expression of CaSR in the HRF+MI/R group gradually increased compared with that in the MI/R and sham groups. The results of the *in vitro* experiments confirmed these findings. In a dose‒response experiment, H/R-injured cells exposed to 5 μM RF for 4 h exhibited increased levels of CaSR, which was accompanied by decreased viability and increased CK and LDH levels.

Many activators of the NLRP3 inflammasome have already been identified; studies have shown that CaSR is generated by NLRP3 activators and acts as a second messenger whose signaling drives inflammasome activation. A previous study reported that CaSR could trigger Ca^2+^ mobilization and NLRP3 activation, which provided new insight for our research 13. Our study showed that the expression of the NLRP3 inflammasome and CaSR was obviously increased and observed throughout the entire cell in the H/R+HRF group, compared with increasing [Ca^2+^]_i_. Moreover, transmission electron microscopy showed that H9c2 cell graduallys became swollen and mitochondria was malformed after H/R, and fragmentation of the cell membrane and swollen mitochondrial structures were observed in the H/R+RF group. These changes were consistent with pyroptosis and indicated that CaSR may be a factor in cell inflammation during HRF-mediated exacerbation of H/R-induced pyroptosis.

**Figure 11:**
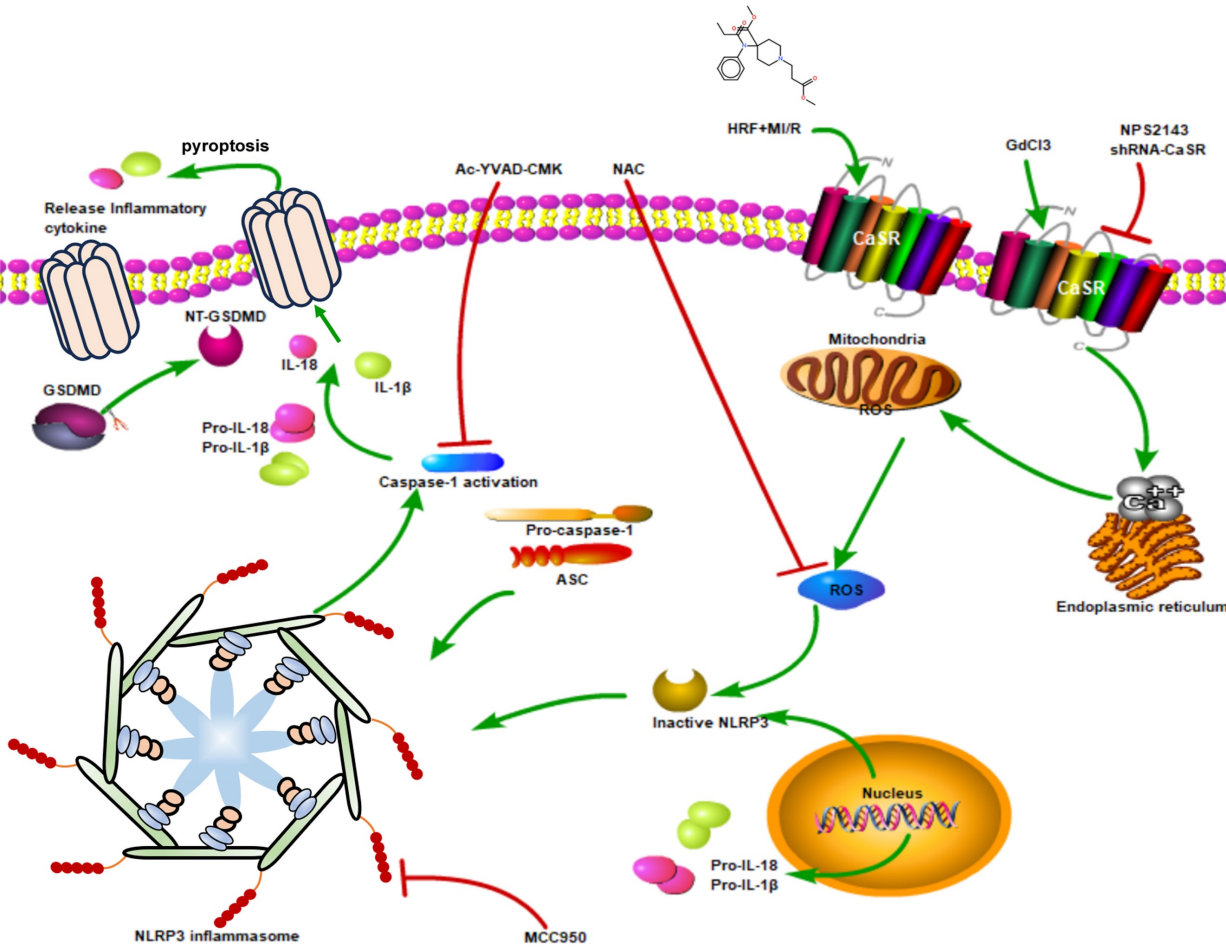
The mechanism that HRF prompts pyroptosis via CaSR/NLRP3/caspase1 signaling pathway under MI/R conditions.

Pyroptosis is a type of programmed cell death that was identified in recent years. Activation of the NLRP3 inflammasome activates caspase-1 and cleaves pro-caspase-1 into activated caspase-1, which then promotes the maturation of the inflammatory cytokines IL-1β and IL-18 and mediates pyroptosis^10^, ^25^. After the cloning of a caspase in apoptosis, caspase-1 has been analyzed mainly in apoptosis studies 26. However, a recent study showed that caspase-1 specifically cleaved the linker between the amino-terminal gasdermin-N and carboxy-terminal gasdermin-C domains in GSDMD, which was required and sufficient for pyroptosis. Cleavage of GSDMD showed intrinsic pyroptosis-inducing activity as a pyroptosis execution protein. A previous study showed that caspase-1-dependent pyroptosis induced by NLRP3 inflammasome activation was involved in myocardial I/R injury 11. In our study, the expression of NLRP3, ASC, GSDMD, pro-caspase 1 (P20), active caspase-1 (P10), IL-1β and IL-18 was increased in the H/R+HRF and GdCl_3_+HRF+H/R groups compared with the H/R group, and this effect was attenuated by NPS2143. We found that the levels of IL-1β and IL-18 in cell supernatants were also increased in the H/R+HRF group and were higher in the GdCl_3_+HRF+H/R group, as was the activity of caspase-1. These results indicated that CaSR promoted HRF-mediated exacerbation of pyroptosis in H9c2 cells under H/R conditions by activating the NLRP3 inflammasome and upregulating the expression of ASC, GSDMD, pro-caspase 1, active caspase-1 (P10), IL-1β and IL-18. Moreover, rescue experiments showed that the CaSR inhibitor NPS2143 can attenuate the inflammatory reaction. To further confirm the role of CaSR-mediated pyroptosis in the cardio-injury induced by HRF, CaSR-shRNA, MCC950, Ac-YVAD-CMK and NAC were applied in the HR+HRF model. We examined cellular apoptosis, ROS and viability, as well as the expression of pyroptosis-related proteins. Our finding that the HRF effects of cardio-injury can be blocked by CaSR-shRNA, MCC950, Ac-YVAD-CMK and NAC and cardio-protection by inhibition of CaSR-mediated pyroptosis is proven. These results also demonstrated that the mechanism of HRF in aggravating MI/R injury was associated with intracellular ROS and that HRF promoted CaSR activation-mediated pyroptosis and inflammatory reactions by increasing Ca^2+^(Figure 11).

However, this study has several limitations that need further exploration. First, CaSR activator and inhibitor were chosen to explore the molecular mechanism of HRF-aggravated MI/R injury *in vitro* experiments, but we did not use any activators or inhibitors *in vivo* experiment as a restriction of the laboratory conditions. Second, this study only investigated the role of CaSR as an upstream factor of the NLRP3 inflammasome and the effects of NLRP3 activation and pyroptosis on HRF-exacerbated injury in an H/R model. Other inflammasomes, AIM2, NLRC4, etc., should be tested to see if NLRP3 is the predominant pathway. Thus, further study is needed to clarify inflammasome activation under H/R+HRF conditions. Third, even though we found that HRF-aggravated MI/R injury was associated with pyroptosis, there are other patterns of cell death in MI/R injury, including apoptosis and ferroptosis. Therefore, we will conduct further evaluation in future studies.

## 5. Conclusions

In summary, our findings demonstrated that exposure to HRF for a short time exacerbates MI/R-induced injury by targeting CaSR to increase cytosolic [Ca^2+^]_i_ and ROS levels, which mediate the NLRP3 inflammasome and activation of the caspase-1 pyroptosis pathway *in vivo* and *in vitro*. Inhibiting CaSR and the NLRP3 inflammasome can mitigate HRF-exacerbated pyroptosis in MI/R, which may be a new strategy to mitigate MI/R-induced injury after high-dose exposure to opioids in the myocardium.

## Abbreviations

CaSR: calcium-sensing receptor
HRF: high-dose remifentanil
MI/R: myocardial ischemia-reperfusion injury
H/R: hypoxia/reoxygenation
GdCl_3_: gadolinium chloride
ROS: reactive oxygenspecies
LDH: lactate dehydrogenase
CK: creatine kinase isoenzyme
NLR: nucleotide-binding domain and leucine-rich repeat-containing
DAMPs: danger-associated molecular patterns
